# Gomisin N Alleviates Ethanol-Induced Liver Injury through Ameliorating Lipid Metabolism and Oxidative Stress

**DOI:** 10.3390/ijms19092601

**Published:** 2018-09-01

**Authors:** Arulkumar Nagappan, Dae Young Jung, Ji-Hyun Kim, Hoyoung Lee, Myeong Ho Jung

**Affiliations:** 1 Division of Longevity and Biofunctional Medicine, School of Korean Medicine, Pusan National University, Yangsan 50612, Korea; arulbiotechtnau@gmail.com (A.N.); dyjung999@naver.com (D.Y.J.); kimji77@pusan.ac.kr (J.-H.K.); 2 Healthy Aging Korean Medical Research Center, School of Korean Medicine, Pusan National University, Yangsan 50612, Korea; 3 KM Fundamental Research Division, Korea Institute of Oriental Medicine, Daejeon 34054, Korea; lhoyoung@kiom.re.kr

**Keywords:** alcoholic liver disease, AMP-activated protein kinase, cytochrome P450 2E1, gomisin N, hepatic steatosis, oxidative stress, sirtuin1

## Abstract

Gomisin N (GN), a lignan derived from *Schisandra chinensis*, has been shown to possess antioxidant, anti-inflammatory, and anticancer properties. In the present study, we investigated the protective effect of GN against ethanol-induced liver injury using in vivo and in vitro experiments. Histopathological examination revealed that GN administration to chronic-binge ethanol exposure mice significantly reduced ethanol-induced hepatic steatosis through reducing lipogenesis gene expression and increasing fatty acid oxidation gene expression, and prevented liver injury by lowering the serum levels of aspartate transaminase and alanine transaminase. Further, it significantly inhibited cytochrome P450 2E1 (CYP2E1) gene expression and enzyme activity, and enhanced antioxidant genes and glutathione level in hepatic tissues, which led to decreased hepatic malondialdehyde levels. It also lowered inflammation gene expression. Finally, GN administration promoted hepatic sirtuin1 (SIRT1)-AMP-activated protein kinase (AMPK) signaling in ethanol-fed mice. Consistent with in vivo data, treatment with GN decreased lipogenesis gene expression and increased fatty acid oxidation gene expression in ethanol-treated HepG2 cells, thereby preventing ethanol-induced triglyceride accumulation. Furthermore, it inhibited reactive oxygen species generation by downregulating CYP2E1 and upregulating antioxidant gene expression, and suppressed inflammatory gene expression. Moreover, GN prevented ethanol-mediated reduction in SIRT1 and phosphorylated AMPK. These findings indicate that GN has therapeutic potential against alcoholic liver disease through inhibiting hepatic steatosis, oxidative stress and inflammation.

## 1. Introduction

Alcohol abuse causes alcoholic liver disease (ALD), which is a major cause of morbidity and mortality worldwide [1]. Chronic alcohol consumption leads to socio-economic and public health problems [2]. Hepatic steatosis, characterized by extensive triglyceride (TG) accumulation, occurs in the initial stages of ALD, and chronic alcohol intake causes hepatic steatosis to progress to the advanced stages, leading to steatohepatitis, fibrosis, cirrhosis, and liver cancer [3]. Ethanol-induced hepatic steatosis is caused by increased de novo lipogenesis, decreased fatty acid oxidation, and the export of very low-density lipoprotein from the liver [3]. In addition, alcohol exposure stimulates lipolysis in the adipose tissue, which increases the influx of fatty acid to the liver [3].

Enhanced lipogenesis is one of the major reasons for the development of hepatic steatosis [4]. Sterol regulatory element-binding protein-1c (SREBP-1c) is an essential transcription factor for de novo lipogenesis, which upregulates downstream lipogenesis genes, including fatty acid synthase (*FAS*), stearoyl CoA desaturase 1 (*SCD1*), acetyl-CoA carboxylase (*ACC*), ATP-citrate lyase (*ACLY*), and diacylglycerol acyltransferase (*DGAT*) [5]. Alcohol exposure stimulates the expression of *SREBP-1c* and its target lipogenesis genes, leading to hepatic steatosis [6]. Decreased fatty acid oxidation also plays an important role in the development of hepatic steatosis [3]. Peroxisome proliferator-activated receptor-α (PPARα) is an important transcription factor that causes fatty acid oxidation by upregulating carnitine palmitoyltransferase 1 (*CPT1*) and acyl-coenzyme A oxidase (*ACO*) [7]. Alcohol exposure downregulates the expression of fatty acid oxidation-related genes, including *PPARα*, *CPT1*, and *ACO* [7].

Reactive oxygen species (ROS) is also a major contributing factor to the pathogenesis of ALD [3]. Alcohol exposure enhances the generation of ROS, including superoxide, hydroxyl radical, and hydrogen peroxide, and suppresses the cellular antioxidant defense system, which leads to oxidative stress in the liver. Several pathways are involved in alcohol-induced oxidative stress [3]. The microsomal cytochrome P450 2E1 (CYP2E1) enzyme catalyzes the conversion of ethanol to acetaldehyde in the presence of iron, and is a major contributor to ROS generation [8]. The expression and activity of CYP2E1 are upregulated due to alcohol exposure, which leads to liver injury [9] and the suppression of CYP2E1 activity reduces the incidence of ethanol-induced fatty liver [10]. Thus, CYP2E1 activation is critical to ALD pathogenesis. Furthermore, alcohol exposure impairs enzymatic and non-enzymatic antioxidant systems that protect hepatocytes against ROS damage [11]. Endogenous antioxidant enzymes involved in ROS elimination include superoxide dismutase (SOD), catalase (CAT), glutathione (GSH)-S-transferase (GST), and glutathione peroxidase (GPX), which are involved in the primary defense against oxidative damage. Non-enzymatic antioxidants, such as GSH as well as vitamins C and E, also play an important role in protecting the cell against lipid peroxidation. Thus, the inhibition of CYP2E1 or the activation of antioxidant systems facilitates the elimination of ROS and protects against ALD.

The fruits of *Schisandra chinensis* have been used for medicinal purposes in Asia [12]. *S. chinensis* extract exhibits a wide spectrum of pharmacological activities, such as anti-aging, anti-melanogenic, anti-inflammatory, and anticancer activities [12]. Previously, we reported that *S. chinensis* extracts having protective effects against endoplasmic stress (ER) stress-induced hepatic steatosis [13]. Numerous types of lignans have been isolated from *S. chinensis* fruits, including gomisin N (GN), which are reported to have hepatoprotective, anticancer, anti-inflammatory and anti-melanogenic activities [14]. Recently, we demonstrated that GN protects against nonalcoholic fatty liver disease (NAFLD) by inhibiting ER stress and activating AMP-activated protein kinase (AMPK) [15,16]. However, the protective effect of GN against ALD and its underlying mechanisms have still not been elucidated. As previously mentioned, TG accumulation, oxidative stress, and inflammation have been implicated in the pathogenesis of ALD, and thus, their inhibition may be a potential treatment strategy against ALD.

Therefore, the present study was designed to investigate the potential protective effects of GN against ethanol-induced liver injury and explore the related mechanisms in a chronic-binge alcohol mouse model and ethanol-treated HepG2 cells. Our findings suggest that GN exerts potential hepatoprotective effects against ethanol-induced liver injury by inhibiting hepatic steatosis, oxidative stress, and inflammation both in vivo and in vitro.

## 2. Results

### 2.1. GN Attenuates Ethanol-Induced Hepatic Steatosis and Liver Injury in Chronic-Binge Ethanol-Fed Mice

**Figure 1:**
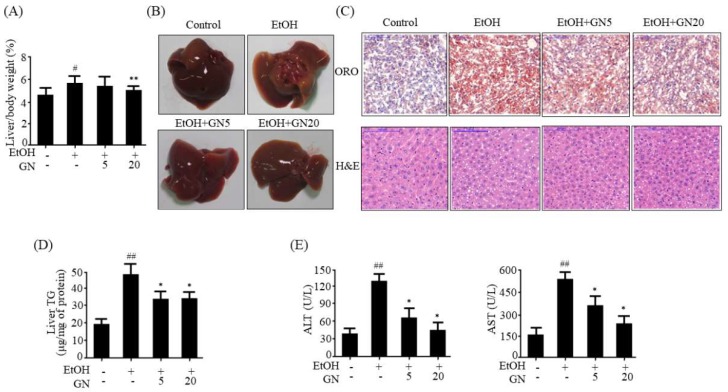
Gomisin N attenuates ethanol-induced hepatic steatosis in chronic-binge ethanol-fed mice. C57BL/6 mice were either pair-fed control or ethanol-containing diet with or without Gomisin N (GN) (5 or 20 mg/kg) for 10 days. (**A**) Ratios of liver-to-body weight, (**B**) Liver morphology. Representative images are shown. (**C**) oil red O (ORO) staining (**upper middle**) and hematoxylin and eosin (H&E) staining (**bottom**) (scale bar = 100 μm). Representative images are shown. (**D**) Hepatic levels of triglyceride (TG). (**E**) Serum levels of alanine aminotransferase (ALT) and aspartate transaminase (AST). Values are the mean ± SD (*n* = 6). ^#^ *p* < 0.05, ^##^ *p* < 0.01 vs. pair-fed control mice, * *p* < 0.05, ** *p* < 0.01 vs. ethanol-fed mice.

Ethanol exposure results in hepatic steatosis [2]. Hepatic de novo lipogenesis is a major contributor to hepatic steatosis [3]. Hence, we first evaluated the effects of GN on chronic-binge alcohol-induced hepatic steatosis. We adopted a chronic-binge alcohol feeding mouse model to establish alcohol-induce steatosis in mice. As shown in Figure 1A, the liver index percentage in the ethanol-fed group significantly increased (5.413 ± 0.534%) when compared with the pair-fed control group (4.393 ± 0.272%), and it was significantly reduced by GN administration, especially high-dose GN (20 mg/kg) (4.892 ± 0.298%). Morphological examination also showed that ethanol-fed mice had white-colored fatty livers, but GN-administered mice had relatively healthy livers (Figure 1B). Additionally, oil red O (ORO) and hematoxylin and eosin (H&E) staining revealed hepatic steatosis (fat deposition) and massive lipid droplets in binge ethanol exposure group compared with the pair-fed control group; however, GN administration significantly reduced ethanol-induced hepatic fat accumulation (Figure 1C). Consistent with these results, hepatic TG accumulation after ethanol feeding was significantly reduced by GN administration (Figure 1D), indicating that GN prevents alcohol-induced hepatic steatosis, thereby reducing liver weight. Serum aspartate transaminase (AST) and alanine aminotransferase (ALT) levels were also measured as they are useful diagnostic indicators of liver damage or hepatotoxicity. Serum ALT and AST levels were significantly elevated in the ethanol-fed group, as compared with the pair-fed control group, and the elevation in both these enzymes were significantly attenuated by GN treatment (Figure 1E).

**Figure 2:**
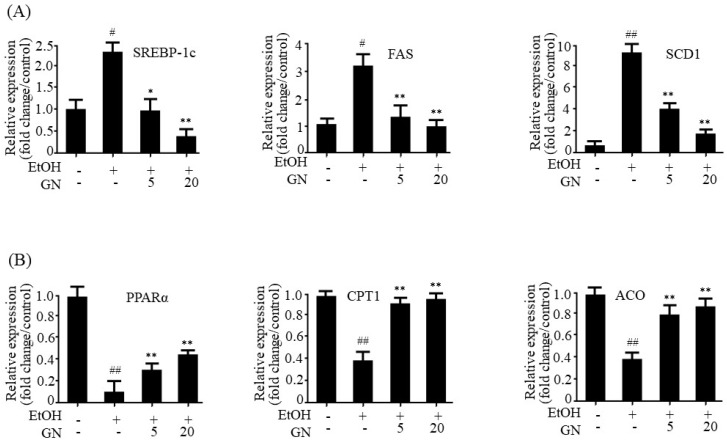
Gomisin N decreases the expression of lipogenesis and increases fatty acid oxidation genes in the liver of chronic-binge ethanol-fed mice. (**A**) qPCR analysis of sterol regulatory element-binding protein-1c (m*SREBP-1c*), fatty acid synthase (m*FAS*), and stearoyl CoA desaturase 1 (m*SCD1*). (**B**) qPCR analysis of peroxisome proliferator-activated receptor-α (m*PPARα*), carnitine palmitoyltransferase 1 (m*CPT1*), and acyl-coenzyme A oxidase (m*ACO*). Values are the mean ± SD (*n* = 6). ^#^ *p* < 0.05, ^##^ *p* < 0.01 vs. pair-fed control mice, * *p* < 0.05, ** *p* < 0.01 vs. ethanol-fed mice.

To further study GN-mediated improvement in hepatic steatosis, we examined the expression of lipogenesis- and fatty acid oxidation-related genes in the liver. As shown in Figure 2A, ethanol feeding significantly increased the mRNA level of SREBP-1c, a key transcription factor for the expression of lipogenesis genes, which was inhibited by GN administration. Consistent with the expression pattern of SREBP-1c, mRNA levels of downstream lipogenesis genes, including *FAS* and *SCD1*, increased in ethanol-fed mice, but GN treatment reduced the expression of lipogenesis genes. In addition to increased de novo lipogenesis, impaired fatty acid oxidation also contributes to hepatic steatosis [3]. Ethanol exposure reduces fatty acid oxidation by downregulating PPARα, an important transcription factor for the expression of fatty acid oxidation genes, which leads to the development of hepatic steatosis [7]. Therefore, we investigated whether GN treatment reverses ethanol-mediated reduction in the expression of fatty acid oxidation genes in binge alcohol-fed mice. As shown in Figure 2B, qPCR results revealed that ethanol exposure reduced mRNA levels of *PPARα*, *CPT1*, and *ACO*, but GN treatment significantly attenuated these ethanol-induced effects. Altogether, these findings suggest that GN could attenuate ethanol-induced steatosis and liver damage in mice.

### 2.2. GN Mitigates Ethanol-Induced Oxidative Stress in Chronic-Binge Ethanol-Fed Mice

**Figure 3:**
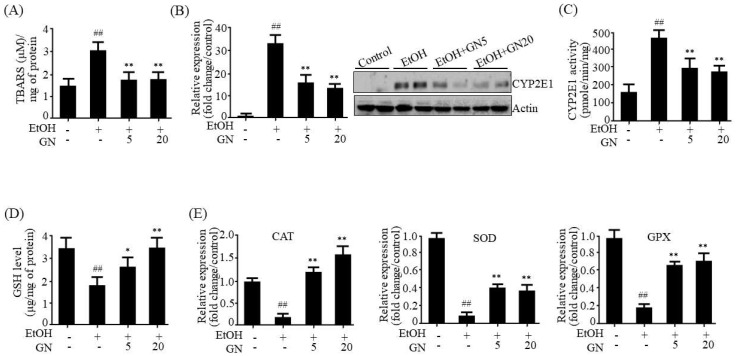
Gomisin N mitigates ethanol-induced oxidative stress in the liver of chronic-binge ethanol-fed mice. (**A**) Hepatic levels of TBARS (malondialdehyde (MDA)). (**B**) qPCR and western blot analyses of cytochrome P450 2E1 (m*CYP2E1*). (**C**) CYP2E1 enzyme activity. (**D**) Hepatic levels of glutathione (GSH). (**E**) qPCR analysis of catalase (m*CAT*), superoxide dismutase (m*SOD*), and glutathione peroxidase (m*GPX*). Values are the mean ± SD (*n* = 6). ^##^ *p* < 0.01 vs. pair-fed control mice, * *p* < 0.05, ** *p* < 0.01 vs. ethanol-fed mice.

Ethanol-induced oxidative stress is a major contributing factor to the pathogenesis of ALD [3]. Therefore, we investigated the protective effects of GN on oxidative stress in the liver of ethanol-fed mice. Malondialdehyde (MDA) has been widely used as an indicator of lipid peroxidation and a marker for oxidative damage. Thus, we determined hepatic MDA level in ethanol-fed mice. As shown in Figure 3A, hepatic MDA level was elevated in ethanol-fed mice, but GN administration attenuated this elevation, suggesting that GN protects against ethanol-induced oxidative stress. CYP2E1, which is induced by ethanol, plays a critical role in ethanol-induced ROS generation [9]. Therefore, we examined whether the GN-mediated protection against oxidative stress is mediated by CYP2E1 inhibition and an improvement in the antioxidant defense system. As shown in Figure 3B, mRNA and protein levels of CYP2E1 were elevated by ethanol feeding, which were reduced by GN administration. Ethanol-induced CYP2E1 enzyme activity was also significantly inhibited by GN administration (Figure 3C). In addition, we examined GSH levels and the expression of antioxidant genes in the liver of ethanol-fed mice. Ethanol feeding significantly reduced both hepatic GSH levels (Figure 3D) and the expression of antioxidant genes, *CAT*, *SOD*, and *GPX* (Figure 3E); however, GN administration inhibited these ethanol-mediated effects. These results demonstrated that GN has protective effects against ethanol-induced oxidative stress, which might contribute to preventing progression of ALD.

### 2.3. GN Prevents Ethanol-Induced Inflammation in Chronic-Binge Ethanol-Fed Mice

**Figure 4:**
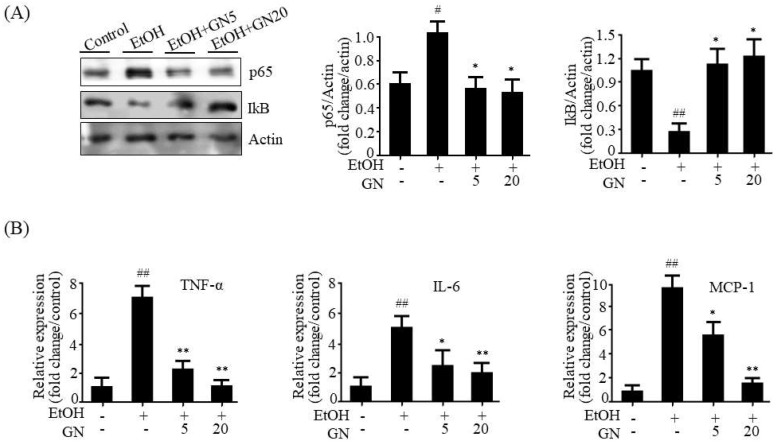
Gomisin N decreases inflammation gene expression by attenuating NF-κB in the liver of chronic-binge ethanol-fed mice. (**A**) Western blot analysis of NF-κB p65 and IκB. Representative images are shown (**left**). Levels of p65 and IκB were quantified by densitometry (**right**). (**B**) qPCR analysis of mTNF-α, mIL-6, and monocyte chemotactic protein-1 (mMCP-1). Values are the mean ± SD (*n* = 6). ^#^ *p* < 0.01, ^##^ *p* < 0.01 vs. pair-fed control mice, * *p* < 0.05, ** *p* < 0.01 vs. ethanol-fed mice.

Inflammation plays a crucial role in the pathogenesis and progression of ALD, particularly the progression of alcoholic hepatic steatosis to steatohepatitis [3]. Thus, the inhibition of ethanol-induced inflammation can prevent the progression of ALD. We investigated whether GN inhibits ethanol-induced inflammation in the liver of ethanol-fed mice. Nuclear factor kappa light chain enhancer of activated B cells (NF-κB) is a key transcription factor that stimulates inflammatory genes, such as tumor necrosis factor-α (*TNF-α*), interleukin-6 (*IL-6*), and monocyte chemotactic protein-1 (*MCP-1*). Therefore, we assessed the inhibitory effect of GN on inflammation in ethanol-fed mice. As shown in Figure 4A, ethanol feeding increased NF-κB p65 protein level, whereas it decreased inhibitor of kappa B (IκB) level. However, GN administration reversed these effects. Consistent with the protective effects of GN on ethanol-induced NF-κB level, GN significantly blocked the ethanol-induced mRNA expression of inflammatory genes, including *TNF-α*, *IL-6*, and *MCP-1* (Figure 4B), indicating that GN exerts anti-inflammatory effects in the liver of ethanol-fed mice.

### 2.4. GN Promoted SIRT1-AMPK Signaling in Chronic-Binge Ethanol-Fed Mice

**Figure 5:**
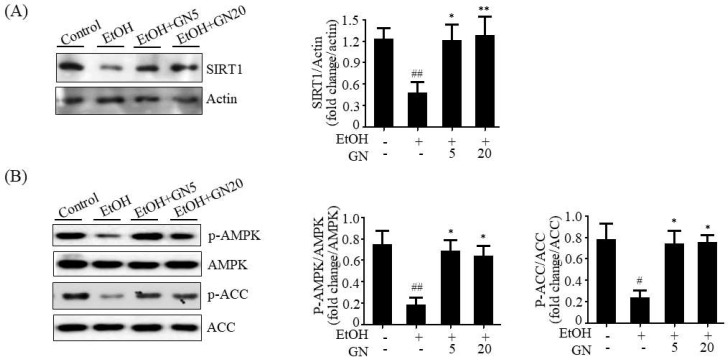
Gomisin N prevents ethanol-mediated reduction in sirtuin1 (SIRT1) and phosphorylated AMP-kinase (AMPK) in the liver of chronic-binge ethanol-fed mice. (**A**) Western blot analysis of SIRT1. Representative images are shown (**left**). SIRT1 level was quantified by densitometry (**right**). (**B**) Western blot analysis of p-AMPK and acetyl-CoA carboxylase (p-ACC). Representative images are shown (**left**). Levels of p-AMPK and p-ACC were quantified by densitometry (**right**). Values are the mean ± SD (*n* = 6). ^#^ *p* < 0.01, ^##^ *p* < 0.01 vs. pair-fed control mice, * *p* < 0.05, ** *p* < 0.01 vs. ethanol-fed mice.

It has been reported that SIRT1, an NAD^+^-dependent histone and protein deacetylase, protects against ALD by regulating lipid metabolism, oxidative stress, and inflammation [17]. Furthermore, previous study has demonstrated that SIRT1 and AMPK regulate each other’s functions [18]. To evaluate whether SIRT1 activation is involved in GN-mediated protection against hepatic steatosis, inflammation, and oxidative stress, we further evaluated the effects of GN on SIRT1-AMPK signaling in the liver of ethanol-fed mice. Compared with pair-fed control mice, ethanol-fed mice exhibited reduced SIRT1 level and AMPK phosphorylation (Figure 5A,B). However, GN administration efficiently restored SIRT1 level and AMPK phosphorylation. These results indicate that GN promotes hepatic SIRT1-AMPK signaling in ethanol-fed mice, which may play an important role in the protection mechanism of GN against ALD.

### 2.5. GN Inhibits Ethanol-Induced Intracellular TG Accumulation in HepG2 Cells

**Figure 6:**
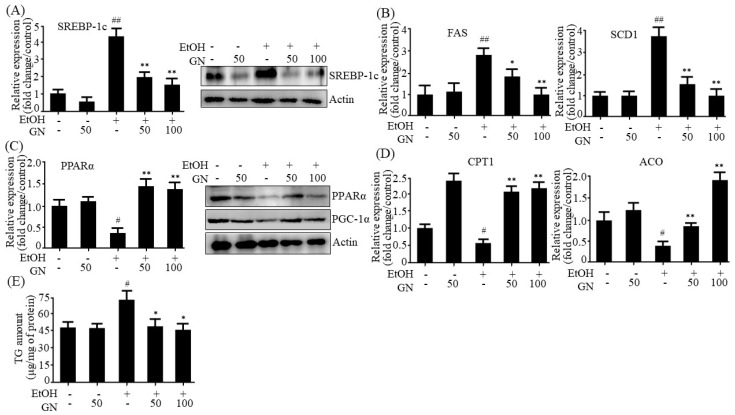
Gomisin N prevents ethanol-induced intracellular TG accumulation in HepG2 cells. HepG2 cells were treated with 50 mM ethanol in the presence or absence of GN (50 or 100 μM) for 24 h. (**A**) qPCR and western blot analysis of hSREBP-1c. (**B**) qPCR analysis of hFAS, and hSCD1. (**C**) qPCR and western blot analysis of hPPARα, western blot analysis of PGC-1α. (**D**) qPCR analysis of hCPT1, and hACO. (**E**) Measurement of intracellular TG levels. Values are the mean ± SD of triplicate experiments. ^#^ *p* < 0.05, ^##^ *p* < 0.01 vs. untreated control, * *p* < 0.05, ** *p* < 0.01 vs. ethanol-treated group.

To observe the inhibitory effect of GN on ethanol-induced steatosis in vitro, we investigated whether GN inhibits the expression of lipogenesis genes in ethanol-treated HepG2 cells. As shown in Figure 6A, ethanol treatment markedly increased mRNA and protein levels of SREBP-1c, which were inhibited by GN pretreatment. Consistent with the expression pattern of SREBP-1c, the mRNA levels of its downstream lipogenesis genes, including *FAS* and *SCD1*, increased in ethanol-treated HepG2 cells (Figure 6B). However, GN pretreatment reduced the expression of lipogenesis genes. Furthermore, we investigated whether GN reverses ethanol-mediated reduction in the expression of fatty acid oxidation genes in ethanol-treated HepG2 cells. As shown in Figure 6C, qPCR and western blotting revealed that ethanol treatment reduced both mRNA and protein levels of PPARα, but GN pretreatment efficiently blocked the reduction. The protein level of peroxisome proliferator activated receptor γ coactivator-1α (PGC-1α), an important coactivator for the expression of fatty acid oxidation genes, was also reduced in ethanol-treated HepG2 cells, but GN pretreatment attenuated the reduction. Moreover, GN pretreatment reversed ethanol-mediated reduction in the expression of fatty acid oxidation genes, such as *CPT1* and *ACO* (Figure 6D).

Then, we assessed the inhibitory effect of GN on intracellular TG accumulation in ethanol-treated HepG2 cells. As shown in Figure 6E, ethanol treatment significantly increased intracellular TG in HepG2 cells, but GN pretreatment significantly suppressed this effect. These results demonstrate that GN prevents ethanol-induced intracellular TG accumulation by downregulating lipogenesis genes and upregulating fatty acid oxidation genes.

### 2.6. GN Inhibits Ethanol-Induced Oxidative Stress in HepG2 Cells

**Figure 7:**
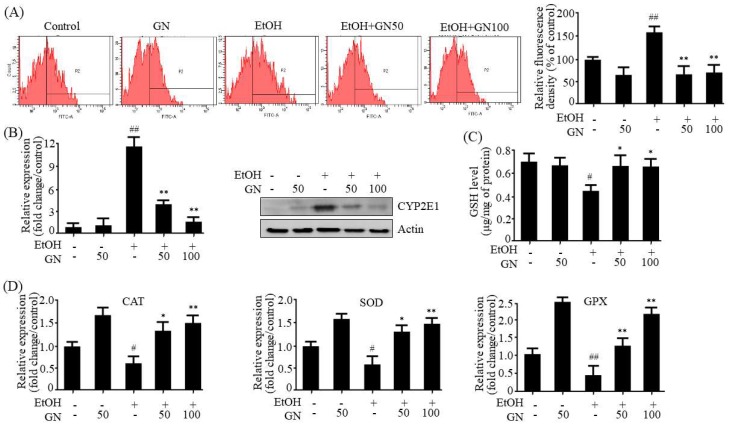
Gomisin N inhibits ethanol-induced oxidative stress in HepG2 cells. HepG2 cells were treated with 50 mM ethanol in the presence or absence of GN (50 or 100 μM) for 24 h. (**A**) 2,7-dichlorofluorescein diacetate (DCFH-DA) analysis of reactive oxygen species (ROS). (**B**) qPCR and western analysis of h*CYP2E1*. (**C**) Measurement of intracellular GSH levels. (**D**) qPCR analysis of h*CAT*, h*SOD*, and h*GPX*. Values are the mean ± SD of triplicate experiments. ^#^ *p* < 0.05, ^##^ *p* < 0.01 vs. untreated control, * *p* < 0.05, ** *p* < 0.01 vs. ethanol-treated group.

To further assess whether GN prevents ethanol-induced oxidative stress, we determined ROS levels in ethanol-treated HepG2 cells. The 2,7-dichlorofluorescein diacetate (DCFH-DA) assay showed that ethanol treatment significantly increased ROS generation, but GN pretreatment markedly inhibited this effect (Figure 7A). Then, we evaluated the inhibitory effects of GN on CYP2E1 expression in ethanol-treated HepG2 cells. As shown in Figure 7B, ethanol treatment increased mRNA and protein levels of CYP2E1; however, GN pretreatment significantly reduced these effects. To confirm the possible mechanism involved in the protective effects of GN against ethanol-induced oxidative stress, we investigated its potential ameliorating effects on the enzymatic and non-enzymatic antioxidant system in ethanol-treated HepG2 cells. Ethanol treatment reduced both GSH levels (Figure 7C) and the expression of antioxidant genes, including *CAT*, *SOD*, and *GPX* (Figure 7D) in HepG2 cells; however, GN pretreatment prevented the ethanol-mediated reduction.

### 2.7. GN Inhibits Ethanol-Induced Inflammation in HepG2 Cells

**Figure 8:**
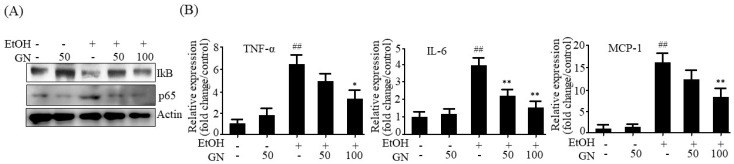
Gomisin N decreases ethanol-induced inflammation gene expression in HepG2 cells. HepG2 cells were treated with 50 mM ethanol in the presence or absence of GN (50 or 100 μM) for 24 h. (**A**) Western blot analysis of IκB and NF-κB p65. (**B**) qPCR analysis of hTNF-α, hIL-6, and hMCP-1. Values are the mean ± SD of triplicate experiments. ^##^ *p* < 0.01 vs. untreated control, * *p* < 0.05, ** *p* < 0.01 vs. ethanol-treated group.

We further investigated whether GN inhibits ethanol-induced inflammation in HepG2 cells. As shown in Figure 8A, ethanol treatment reduced IκB level and increased NF-κB p65 level; however, GN pretreatment reversed these effects, indicating that GN inhibits ethanol-induced NF-κB activation. Additionally, GN pretreatment reduced ethanol-induced mRNA levels of NF-κB target inflammatory genes, including *TNF-α*, *IL-6*, and *MCP-1* (Figure 8B), which indicates that GN inhibits ethanol-induced inflammation.

### 2.8. GN Upregulates SIRT1-AMPK Pathway in Ethanol-Treated HepG2 Cells

**Figure 9:**
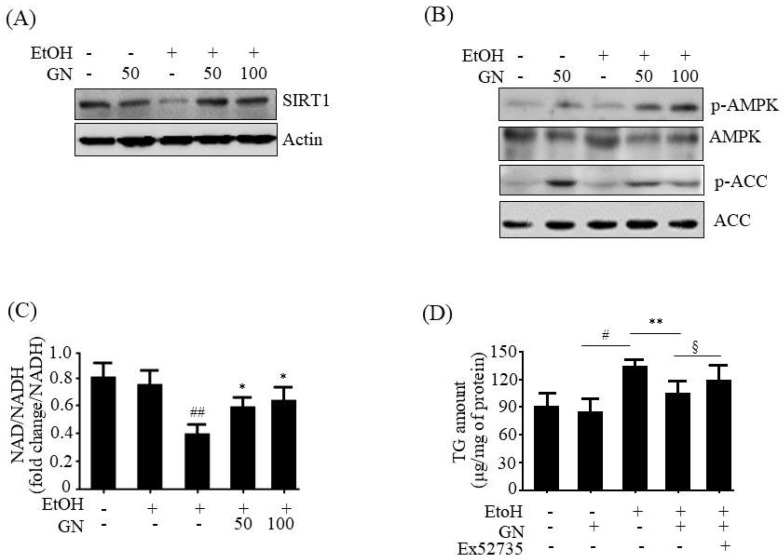
Gomisin N reverses ethanol-mediated reduction in SIRT1 and phosphorylated AMPK in HepG2 cells. HepG2 cells were treated with 50 mM ethanol in the presence or absence of GN (50 or 100 μM) for 24 h. (**A**) Western blot analysis of SIRT1. (**B**) Western blot analysis of p-AMPK and p-ACC. (**C**) Measurement of NAD^+^/NADH ratio. (**D**) HepG2 cells were pretreated with GN (100 μM) for 3 h or with Ex52735 (10 μM) for 6 h and then treated with ethanol (100 μM). Measurement of intracellular TG levels. Values are the mean ± SD of triplicate experiments. ^#^ *p* < 0.05, ^##^ *p* < 0.01 vs. untreated control, * *p* < 0.05, ** *p* < 0.01 vs. ethanol-treated group. ^§^ *p* < 0.05 vs. ethanol and GN-treated group.

To further evaluate whether SIRT1 activation is involved in GN-mediated protection against liver injury, we examined SIRT1 expression in ethanol-treated HepG2 cells. As shown in Figure 9A, ethanol treatment resulted in decreased SIRT1 protein level, but GN pretreatment significantly blocked the reduction in SIRT1 level. Additionally, we investigated the effects of GN on AMPK activation in ethanol-treated HepG2 cells. Western blotting showed that ethanol exposure reduced the phosphorylation of AMPK and its downstream *ACC*, which was reversed by GN pretreatment (Figure 9B). AMPK activates SIRT1 by increasing intracellular NAD^+^ level because NAD^+^ is a co-substrate for SIRT1 activity [19]. Thus, we measured intracellular NAD^+^/NADH ratio in ethanol-treated HepG2 cells. As shown in Figure 9C, ethanol treatment significantly decreased the ratio of NAD^+^/NADH; however, GN pretreatment reversed ethanol-mediated reduction in NAD^+^/NADH ratio. Collectively, these results indicate that GN upregulates SIRT1-AMPK signaling in ethanol-treated HepG2 cells. To further evaluate whether SIRT1 plays a role in GN-mediated protection against steatosis, HepG2 cells were pretreated with GN and/or the specific SIRT1 antagonist Ex52735 before treatment with ethanol, and TG accumulation was accessed. As shown in Figure 9D, GN pretreatment reversed ethanol-induced intracellular TG accumulation in HepG2 cells. However, co-treatment with Ex52735 significantly blocked the GN-mediated reduction in TG accumulation, suggesting that GN-induced protection against ethanol-induced hepatic steatosis is mediated by SIRT1 up-regulation.

## 3. Discussion

Although ALD causes an economic burden and has serious effects on public health, there are few satisfactory therapeutic approaches to prevent or treat the progression of ALD [4]. To date, numerous studies have attempted to discover bioactive components from herbal sources for the prevention and treatment of ALD [20]. Previously, we reported that GN, a phytochemical derived from *S. chinensis*, inhibits high-fat diet-induced hepatic steatosis by inhibiting endoplasmic reticulum stress and activating AMPK [15,16]. In the present study, we investigated whether GN also prevents ALD. We demonstrated that GN administration protected against ethanol-induced hepatic steatosis, oxidative stress, and inflammation in chronic-binge alcohol feeding mice. Consistent with in vivo results, GN downregulated lipogenesis genes and upregulated fatty acid oxidation genes in ethanol-treated HepG2 cells, thereby reducing intracellular TG accumulation. Furthermore, GN suppressed ethanol-induced ROS production and inflammation. GN reversed ethanol-mediated inhibition of SIRT1 and AMPK in both mice and HepG2 cells, and inhibited the expression and activity of CYP2E1.

The pathogenesis of ALD is associated with increased hepatic TG accumulation, oxidative stress, and inflammatory cytokines [3]. Hepatic steatosis is the earliest stage of the broad spectrum of conditions that constitute ALD, and continuous alcohol intake causes a progression to the advanced disease stage due to oxidative stress and inflammation [3]. Therefore, we hypothesized that the inhibition of TG accumulation, oxidative stress, and inflammation might be a potential strategy to prevent the progression of ALD. Hepatic TG accumulation is caused by enhanced de novo lipogenesis and impaired fatty acid oxidation [3]. Alcohol intake stimulates lipogenesis by upregulating SREBP-1c and its downstream lipogenesis genes [6], whereas it lowers fatty acid oxidation by downregulating PPARα-PGC-1α and its target fatty acid oxidation genes [7]. Hence, we assessed the protective effects of GN against ethanol-induced hepatic steatosis in chronic-binge alcohol-fed mice and HepG2 cells. Our results showed that ethanol feeding induces hepatic steatosis, as observed by the measurement of hepatic TG accumulation and histological examination. However, GN administration efficiently blocked hepatic TG accumulation and converted the white-colored fatty liver into a relatively healthy liver. Moreover, GN administration reduced ethanol-induced serum levels of ALT and AST, indicating that GN ameliorates ethanol-induced hepatic injury and toxicity by attenuating hepatic steatosis. Furthermore, we observed that GN administration decreases the ethanol-induced expression of lipogenesis genes in ethanol-fed mice liver and HepG2 cells, but increases the expression of fatty acid oxidation genes, indicating that GN prevents ethanol-induced hepatic steatosis by decreasing lipogenesis and increasing fatty acid oxidation both in vivo and in vitro.

Sirtuins are a group of highly conserved NAD^+^-dependent histone and protein deacetylases and/or ADP-ribosyl transferases that play important functions in numerous biological processes by regulating the transcriptional activation and/or gene expression of several crucial transcription factors and transcription co-activators that are involved in metabolic homeostasis [17]. SIRT1 inhibits hepatic de novo lipogenesis by deacetylating lipogenesis transcription factors, such as SREBP-1c and carbohydrate-responsive element-binding protein (ChREBP), stimulates fatty acid β-oxidation by deacetylating the fatty acid oxidation transcription factor and coactivator PPARα/PGC-1α, inhibits hepatic oxidative stress by enhancing the antioxidant system, and reduces inflammation by deacetylating NF-κB in the liver. Recent study demonstrated that SIRT1 activation could protect against alcohol-induced liver diseases [17]. Furthermore, SIRT1 and AMPK regulate each other’s functions. SIRT1 can stimulate AMPK by increasing cellular NAD^+^ levels that is used as a substrate for SIRT1 activity [18,19]. In this regard, we investigated whether SIRT1-AMPK activation is associated with the beneficial protective effect of GN against ALD. Ethanol exposure significantly suppressed the levels of SIRT1 and phosphorylated AMPK in the liver of ethanol-fed mice and HepG2 cells, but GN administration blocked this reduction. In addition, we examined the effects of GN on cellular NAD^+^ level in ethanol-treated HepG2 cells because NAD is a stimulator for SIRT1 activity. Ethanol treatment resulted in decreased NAD^+^/NADH ratio; however, pretreatment with GN reversed the reduction. In consistent with this result, GN recovered NAD/NADH ratio in SK-Hep1 cells (Figure S1). Moreover, the inhibition of SIRT1 using a SIRT1 inhibitor significantly prevented ethanol-induced cellular TG accumulation in HepG2 cells. Based on these results, although we have not measured the acetylated SREBP and PPARα/PGC-1α levels, GN-mediated SIRT1 activation might contribute to inhibition of lipogenesis and stimulation of fatty acid oxidation, thereby leading to decreased TG accumulation. To provide evidence for the involvement of SIRT1 in TG accumulation, the acetylated SREBP and PPARα/PGC-1α levels should be determined in further study. AMPK also plays a key role in the regulation of lipid and glucose metabolism [21]. AMPK can phosphorylate SREBP-1c and inhibit its transcriptional activity, thereby decreasing lipogenesis. In contrast, AMPK stimulates fatty acid oxidation by upregulating fatty acid oxidation genes and inactivating ACC1 that catalyzes the synthesis of malonyl-CoA, a potent inhibitor of CPT1. Our present results showed that GN treatment efficiently enhanced AMPK phosphorylation in the liver of chronic-binge alcohol feeding mice and ethanol-treated HepG2 cells, which was consistent with reduced lipogenesis genes expression and increased fatty acid oxidation genes expression, suggesting that AMPK activation might also participate in GN-mediated inhibition of TG accumulation. Taken together, these results suggest that the SIRT1-AMPK pathway might play a critical role in the protection of GN against ethanol-induced hepatic steatosis.

Oxidative stress is also a contributing factor to ethanol-induced ALD pathogenesis [3]. Alcohol exposure increases the generation of ROS. Among the several pathways involved in ROS generation by alcohol, CYP2E1 is considered to be the major contributor to ethanol-induced ROS generation [10]. The expression and activity of CYP2E1 are enhanced due to alcohol intake and cause liver injury. Therefore, the inhibition of CYP2E1 expression could effectively block the progression of ALD. In the present study, we evaluated the inhibitory effects of GN on ethanol-induced CYP2E1 expression and subsequent ROS generation in both chronic-binge alcohol feeding mice and ethanol-treated HepG2 cells. Alcohol feeding significantly elevated the expression and activity of CYP2E1 in the liver of chronic-binge alcohol-fed mice and subsequently, increased hepatic MDA levels. However, GN administration markedly reversed these ethanol-induced effects. Ethanol treatment also elevated CYP2E1 expression and increased ROS production in HepG2; however, GN treatment reduced these ethanol-induced effects in HepG2 cells. These results suggest that GN might exert protective effects against ethanol-induced oxidative stress through inhibition of CYP2E1. Furthermore, alcohol exposure impairs enzymatic and non-enzymatic antioxidant systems that protect hepatocytes against ROS damage [11]. Therefore, we investigated whether GN exerts antioxidant effects by reversing the ethanol-mediated impairment of the antioxidant system. The present results showed that binge alcohol feeding in mice and ethanol treatment in HepG2 cells not only repressed the expression of antioxidant genes, but also reduced GSH levels. However, GN administration reversed these ethanol-mediated effects, indicating that GN might also protect against ethanol-induced ROS generation by upregulating antioxidant systems. SIRT1 stimulates antioxidant genes expression via nuclear factor E2-related factor 2 (Nrf-2) activation [22] or PGC-1α deacetylation [23], and inhibits oxidative stress. Based on the present results that GN treatment upregulated antioxidant genes expression in the liver of GN-treated mice and HepG2 cells concomitant with SIRT1 activation, SIRT1 might also play a role in GN-mediated inhibition of ROS generation through upregulation of antioxidant defense.

Furthermore, inflammation also plays a crucial role in the pathogenesis of ALD [3]. Our present results showed that ethanol exposure significantly upregulates inflammation gene expression in the liver of chronic-binge alcohol feeding mice and in ethanol-treated HepG2 cells. However, these effects were efficiently reversed by GN administration through inhibiting NF-κB activation. ROS can activate transcription factors such as NF-κB and activator protein 1 (AP-1), and induce inflammatory gene expression [24]. The present study revealed that GN inhibited ethanol-induced ROS generation and NF-κB activation consistent with repression of inflammatory genes expression. These results suggest that GN-mediated inhibition of ROS generation might lead to repress inflammation through attenuation of NF-κB. Furthermore, SIRT1 can deacetylate NF-κB and inhibit its transcriptional activity, thereby reducing inflammatory genes expression. Therefore, SIRT1 activation might also play a role in GN-mediated suppression of inflammation. In addition, GN reduced ethanol-induced inflammation gene expression in SK-Hep1 cells (Figure S2). Taken together, our present study demonstrated that GN inhibits ethanol-induced inflammation, which might prevent the progression of ALD.

**Figure 10:**
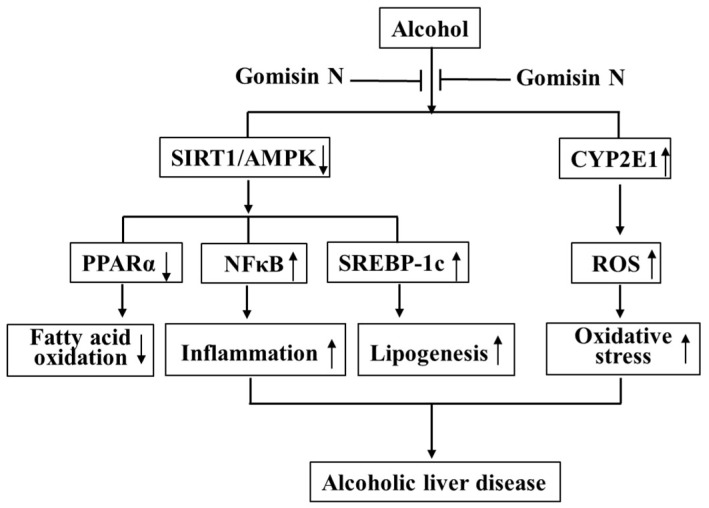
Schematic representation of the proposed mechanism by which GN protects against alcoholic liver disease (ALD) by inhibiting hepatic steatosis, oxidative stress, and inflammation. GN exerts potential hepatoprotective effects against ethanol-induced liver injury by activating SIRT1/AMPK and inhibiting CYP2E1. ↑ means upregulation and ↓ means downregulation

In conclusion, GN exerted a protective effect against ALD by inhibiting hepatic steatosis, oxidative stress, and inflammation both in vivo and in vitro. SIRT-AMPK activation and CYP2E1 inhibition might play a critical role in GN-mediated protection against ALD (Figure 10). Our previous study also demonstrated the protective effect of GN against NAFLD [10]. Altogether, GN could be a potential therapeutic agent for preventing both ALD and NAFLD, and conducting further clinical trials might be worthwhile.

## 4. Materials and Methods

### 4.1. Reagents

GN (≥98% purity) was obtained from ChemFaces (Wuhan, China). Antibodies against SREBP-1c (H-160; sc-8984), PPARα (H-2; sc-398394), PGC-1α (H-300; sc-13067), IκBα (H-4; sc-164), NF-κB p65 (F-6; sc-8008p65), SIRT1 (H-300; sc-15404), AMPK α1/2 (D-6; sc-74461), and β-actin (C4; sc-47778) were purchased from Santa Cruz Biotechnology (Santa Cruz, CA, USA). Specific primary antibodies against p-ACC (Ser79; #3661s) and ACC (C83B10; #3676s) were purchased from Cell Signaling Technology (Danvers, MA, USA). Antibodies against CYP2E1 (ab28146) and p-AMPK α1 (Thr172; PA5-17831) were purchased from Abcam (Cambridge, MA, USA) and Invitrogen (Rockford, IL, USA), respectively. Ethanol and Ex52735 were obtained from Sigma (St. Louis, MO, USA).

### 4.2. Animal Experiments

All animal experiments were approved by Pusan National University’s Institutional Animal Care and Use Committee, in accordance with the established ethical and scientific care procedures (PNU-2017-1631: 28 July 2017). Male C57BL/6N mice (8-week-old, 20–22 g) were purchased from Doo Yeol Biotech (Seoul, South Korea). The mice were housed in a pathogen-free laboratory environment, maintained at 22 ± 2 °C temperature and 50–60% relative humidity, with a 12-h light-dark cycle throughout the experiment. All mice were randomly divided into 4 groups as follows: normal group (*n* = 10), ethanol group (*n* = 10), ethanol + GN-5 mg/kg (*n* = 11) group, and ethanol + GN-20 mg/kg (*n* = 11) group. Mice were fed according to the NIAAA model (chronic-binge) as described previously [25]. The mice were initially fed the control Lieber-DeCarli liquid diet and ethanol Lieber-DeCarli diet (gradually increasing 0–5% ethanol concentrations) (Research Diets Inc., New Brunswick, NJ, USA) ad libitum for the first 5 days for acclimatization. Then, the feeding protocol was switched to chronic-binge feeding, in which mice were allowed free access to the ethanol Lieber-DeCarli diet containing 5% (*v*/*v*) ethanol for 10 days, and control-fed mice were pair-fed with an isocaloric control diet. GN-treated mice were orally administered daily 5 or 20 mg/kg of GN. On the 11th day, mice in ethanol groups were orally administered a single dose of ethanol (5 g/kg), and mice in control groups were orally administered dextrin maltose in the early morning. Further, the mice were euthanized, and blood and tissue samples were collected 9 h post oral administration. Isolated liver tissues were immediately frozen in liquid nitrogen and stored at −80 °C until further analyses.

### 4.3. Histopathological Analysis

Isolated liver tissues were dissected, fixed in 10% buffered formalin, embedded in paraffin, and then 5-μm sections were cut using a frozen microtome (HM560H, Microm International, Walldorf, Germany). The liver tissue sections were stained with hematoxylin and eosin (H&E) and then with oil red O (ORO); stained sections were then observed under a light microscope.

### 4.4. TG Measurement

HepG2 cell suspensions were mixed with 750 μL of chloroform/methanol/H2O (8:4:3, *v*/*v*/*v*) to extract TG. The cell suspensions were incubated at room temperature for 1 h and centrifuged at 800× *g* for 10 min. The bottom layer (organic phase) obtained was dried overnight, dissolved in ethanol, and then an enzyme reaction kit (Asan Pharmaceutical, Seoul, South Korea) was used to measure TG concentration, which was normalized to the protein concentration.

### 4.5. Biochemical Analysis

Blood samples were collected and centrifuged at 1000× *g* for 15 min at 4 °C to obtain serum samples, which were stored at −80 °C until analyses. Serum alanine aminotransferase (ALT) and aspartate aminotransferase (AST) were determined by using FUJI DRI-CHEM 7000i (FUJI FILM, Tokyo, Japan).

### 4.6. Measurement of Hepatic Lipid Peroxidation

Hepatic lipid peroxidation was expressed in terms of malondialdehyde (MDA) concentration that was measured by the thiobarbituric acid reactive substances (TBARS) method using a commercially available kit (OxiSelect™ TBARS assay kit-MDA Quantitation, Cell Biolabs Inc., San Diego, CA, USA) according to the manufacturer’s instructions. Briefly, the liver tissue was homogenized in PBS containing 1× butylated hydroxytoluene solution on ice. After centrifugation at 10,000× *g* for 5 min at 4 °C, the supernatant was collected for the measurement of TBARS level. Absorbance was measured at 532 nm using a microplate reader.

### 4.7. Measurement of CYP2E1 Enzyme Activity

Mouse liver microsomes were prepared as described previously [26]. Briefly, the liver tissues were homogenized in 125 mM KCl and 10 mM KPi buffer (pH 7.4), and centrifuged at 600× *g* for 10 min. The supernatant was removed and centrifuged twice at 8500× *g* for 10 min. Finally, the supernatant was centrifuged at 100,000× *g* for 60 min and the pellet containing the microsomes was obtained. The pellet was resuspended in 125 mM KCl-10 mM KPi (pH 7.4) and the protein concentration was determined using the Bio-Rad DC Protein Assay Kit (Bio-Rad, Hercules, CA, USA).

Microsomal CYP2E1 (p-nitrophenol-2-hydroxylase) activity was assayed as described previously [27]. Briefly, the microsomes were incubated in an assay buffer containing KPi buffer (50 mM, pH 7.4), NADPH (1 mM), and p-nitrophenol (0.1 mM) at 37 °C using a shaking water bath. After 30 min, the reaction was stopped by the addition of 500 μL of 0.75 N perchloride acid, and centrifuged at 5000× *g* for 45 min. Subsequently, 250 μL of 2 M NaOH was added to the supernatant, and the absorbance was measured at 546 nM spectrophotometrically.

### 4.8. Measurement of Liver GSH Level

GSH level in the liver was measured with a commercially available kit (K261-100, BioVision, CA, USA) following the manufacturer’s protocol. Briefly, liver tissues were homogenized with cold GSH assay buffer, and then centrifuged for 10 min at 8000× *g*. The supernatant was collected and GSH level was measured based on the absorbance at 405 nm using a microplate reader.

### 4.9. Cell Culture

The HepG2 and SK-HEP-1 cells were obtained from American Type Culture Collection (Manassas, VA, USA) and grown in DMEM supplemented with 10% heat-inactivated FBS, penicillin (100 units/mL), and streptomycin sulfate (100 μg/mL) at 37 °C in an atmosphere of 5% CO_2_.

### 4.10. Quantitative Polymerase Chain Reaction (qPCR)

Total RNA was isolated from mouse livers or HepG2 cells using TRIzolTM (Invitrogen). Isolated RNA (1 µg) was reverse-transcribed using TOPScript RT DryMix (Enzynomics, Daejeon, Korea). Quantitative real-time PCR was performed using a SYBR Green premixed Taq reaction mixture with gene-specific primers as listed in Table S1.

### 4.11. Western Blot Analysis

Protein lysates from liver tissues or HepG2 cells were prepared using Pro-Prep Protein Extraction Solution (Intron Biotechnology, Seoul, Korea) according to the manufacturer’s instructions. The protein lysates (50 μg) were subjected to SDS-PAGE and then transferred to polyvinylidene difluoride membranes (Amersham Pharmacia Biotech, Amersham, UK). The membranes were incubated with specific primary antibodies (1:1000) at 4 °C overnight followed by incubation with anti-rabbit or anti-mouse secondary antibodies (1:1000) (Santa Cruz Biotechnology) conjugated to peroxidase, and protein bands were visualized using an enhanced chemiluminescence system (ECL Advance, GE Healthcare, Hatfield, UK).

### 4.12. Determination of Intracellular ROS Levels

HepG2 cells were treated with ethanol (50 mM) for 3 h after incubating with GN (50 μM) for 3 h, and the ROS level was measured using the fluorescent dye, 2,7-dichlorofluorescein diacetate (DCFH-DA, Sigma) by flow cytometry with the FACSCantoII (BD Biosciences, San Jose, CA, USA). Briefly, HepG2 cells were suspended in PBS with DCFH-DA (10 μM) at 37  °C for 30 min. Then, the cells were harvested and fluorescence was determined using the FACS Diva 6.3.1 software (BD Biosciences).

### 4.13. NAD^+^/NADH Ratio Measurement

The NAD^+^/NADH ratio in HepG2 cells was measured with the NAD^+^/NADH quantification kit (K377-100; Biovision) based on an enzymatic cycling reaction, according to the manufacturer’s instructions.

### 4.14. Statistical Analysis

All data are presented as the mean ± SD. The data were analyzed using one-way ANOVA, and the differences between means were determined using the Tukey–Kramer post hoc test. Values were considered statistically significant at *p* < 0.05.

## Supplementary Materials

Supplementary materials can be found at http://www.mdpi.com/1422-0067/19/9/2601/s1.

## Abbreviations

ALD	Alcoholic liver disease
CYP2E1	Cytochrome P450 2E1
GN	Gomisin N
ROS	Reactive oxygen species
SIRT1	Sirtuin 1
SREBP-1c	Sterol regulatory element-binding protein-1c
TG	Triglyceride
