# Supplementary material for: Gomisin N Alleviates Ethanol-Induced Liver Injury through Ameliorating Lipid Metabolism and Oxidative Stress
Source: Int J Mol Sci. 2018 Sep 1;19(9):2601. doi: 10.3390/ijms19092601 (PMC6164513; doi:10.3390/ijms19092601)
Supplement: Supplementary file 1 [file ijms-19-02601-s001.pdf]

Supplementary Table S1. qPCR primers list

Human

Gene	Forward primer	Reverse primer
hSREBP1c	CGGAGCCATGGATTGCACT	TAGGCCAGGGAAGTCACTG
hCYP2E1	AGCAGATTTCTGGGAGCCTCAGTT	AGTGGAACTGTGACTTGGAGCTGT
hFAS	TCGTGGGCTACAGCATGGT	GCCCTCTGAAGTCGAAGAAGAA
hSCD1	CCAGTCAACTCCTCGCACTT	AGCCAGGTTTGTAGTACCTCC
hPPARα	GGACAGCAAATCTTGAAGCAGC	CTCTGATCCCTCTAGCACCTT
hCPT1	TTTCCTTGCTGAGGTGCTCT	TCTCGCCTGCAATCATGTAG
hACO	TCCTGCCCACCTTGCTICAC	TTGGGGCCGATGTCACCAAC
hCAT	GAACTGTCCCTACCGTGCTCGA	CCAGAATATTGGATGCTGTGCTCCAGG
hSOD	AATGGACCAGTGAAGGTGTGGGG	CACATTGCCCAAGTCTCCAACATGC
hGPX	CGGCCCAGTCGGTGTATGC	CGTGGTGCCTCAGAGGGAC
hTNF-α	TGCTTGTTCCTCAGCCTCTT	ATGGGCTACAGGCTTGTCACT
hIL-6	ACTCACCTCTTCAGAACGAATTG	CCATCTTTGGAAGGTTCAGGTTG
hMCP-1	CCCCAGTCACCTGCTGTTAT	TGGAATCCTGAACCCACTTC
hm18s	CGGCTACCACATCCAAGGAA	GCTGGAATTACCGCGGCT

Mouse

Gene	Forward primer	Reverse primer
mSREBP1c	GGAGCCATGGATTGCACATT	GGCCCGGGAAGTCACTGT
mCYP2E1	CTTTGCAGGAACAGAGACCA	ATGCACTACAGCGTCCATGT
mFAS	AGG TGG TGA TAG CCG GTA TGT	TGG GTA ATC CAT AGA GCC CAG
mSCD1	CTGTACGGGATCATACTGGTTC	GCCGTGCCTTGTAAGTTCTG
mPPARα	AGCTGGTGTAGCAAGTGT	TCTGCTTTCAGTTTTGCTTT
mCPT1	ACTCCTGGAAGAAGAAGTTCA	AGTATCTTTGACAGCTGGGAC
mACO	TGTTAAGAAGAGTGCCACCAT	ATCCATCTCTTCATAACCAAATTT
mCAT	TGAGAAGCCTAAGAACGCAATTC	\CCCTTCGCAGCCATGTG
mSOD	CCAGTGCAGGACCTCATTTT	GTTTACTGCGCAATCCCAAT
mGPX	CCACCGTGTATGCCTTCTCC	GATCGTGGTGCCTCAGAGAG
mTNF-α	CCCTCACACTCAGATCATCTTCT	GCTACGACGTGGGCTACAG
mIL-6	TAGTCCTTCCTACCCCAATTTCC	TTGGTCCTTAGCCACTCCTTC
mMCP-1	GCATCCACGTGTTGGCTCA	CTCCAGCCTACTCATTGGGATCA
hm18s	CGGCTACCACATCCAAGGAA	GCTGGAATTACCGCGGCT

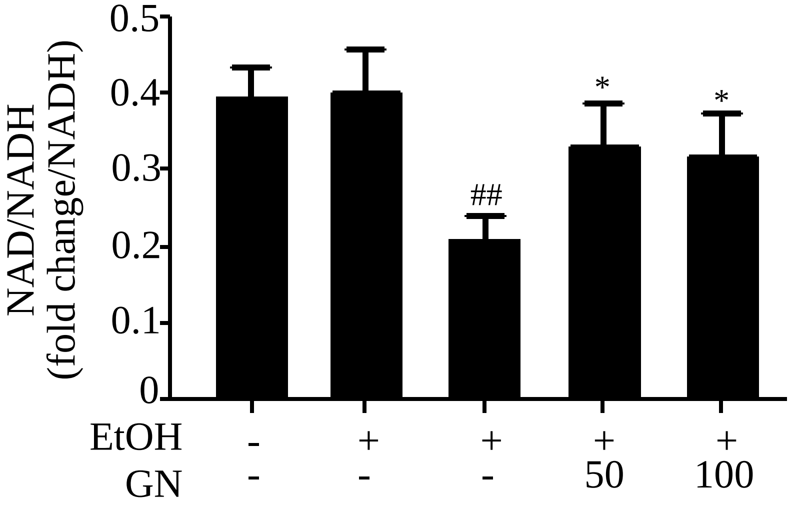

Supplementary Figure S1. Gomisin N reverses ethanol-mediated reduction in NAD^+/NADH ratio in SK-HEP-1 cells. SK-HEP-1 cells were treated with 50 mM ethanol in the presence or absence of GN (50 μM or 100 μM) for 24 h. The NAD^+/NADH ratio in SK-HEP-1 cells was measured with the NAD^+/NADH quantification kit according to the manufacturer's instructions. Values are the mean±SD of triplicate experiments. $^{\#\#}p<0.01$ vs. untreated control, $^{*}p<0.05$ vs. ethanol-treated group.

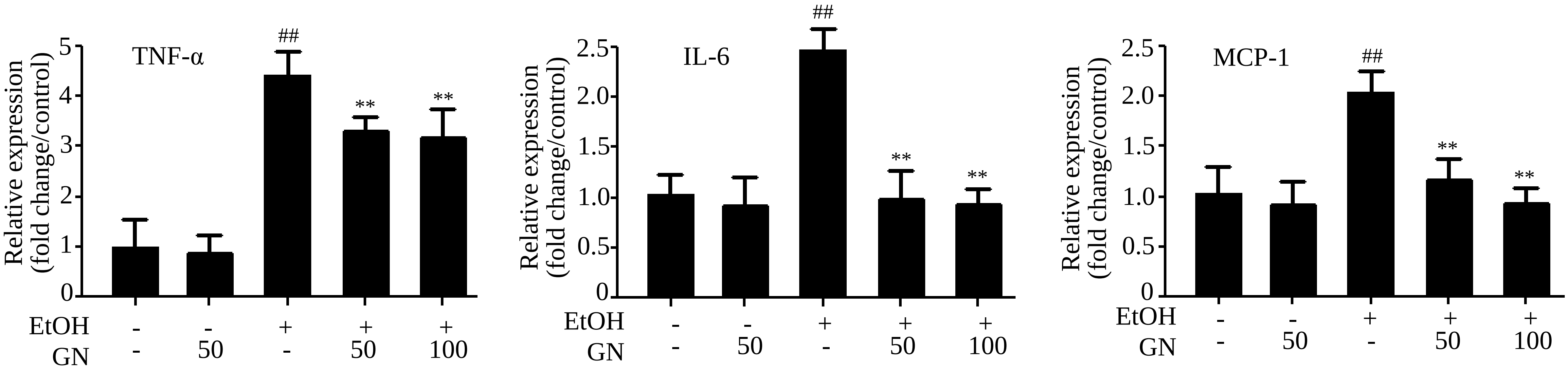

Supplementary Figure S2. Gomisin N decreases ethanol-induced inflammation gene expression in SK-HEP-1 cells. SK-HEP-1 cells were treated with 50 mM ethanol in the presence or absence of GN (50 μM or 100 μM) for 24 h. The expressions of h*TNF-α*, h*IL-6*, and h*MCP-1* were analysed by qPCR. Values are the mean±SD of triplicate experiments. $^{\#\#}p<0.01$ vs. untreated control, $^{**}p<0.01$ vs. ethanol-treated group.
